# Supplementary material for: Influence of Vitamin D Status and Supplementation on Metabolomic Profiles of Older Adults
Source: Metabolites. 2023 Jan 23;13(2):166. doi: 10.3390/metabo13020166 (PMC9961282; doi:10.3390/metabo13020166)
Supplement: Supplementary file 1 [file metabolites-13-00166-s001.zip › metabolites-2019323-supplementary.pdf]

Supplementary Table 1. Metabolites measured and quantified in baseline serum samples

Metabolite Class	Metabolite (μM)	Mean	SE
Amino acid	Glycine	211.217	5.788
Amino acid	Alanine	363.096	7.359
Amino acid	Serine	102.546	1.835
Amino acid	Proline	147.777	4.580
Amino acid	Valine	214.145	3.737
Amino acid	Threonine	99.557	2.435
Amino acid	Citrulline	34.110	0.954
Amino acid	Tyrosine	77.135	1.658
Amino acid	Tryptophan	54.061	1.010
Amino acid	Leucine	111.354	2.511
Amino acid	Isoleucine	59.022	1.401
Amino acid	Asparagine	45.574	0.732
Amino acid	Aspartic acid	8.764	0.211
Amino acid	Glutamine	574.337	7.879
Amino acid	Glutamic acid	39.864	2.473
Amino acid	Methionine	22.018	0.369
Amino acid	Histidine	78.219	0.888
Amino acid	Ornithine	62.427	1.244
Amino acid	Lysine	206.217	3.208
Amino acid	Phenylalanine	62.081	0.862
Amino acid	Methionine-sulfoxide	0.747	0.016
Amino acid	Arginine	108.319	1.869
Amino acid	Homocysteine	8.058	0.111
Biogenic Amine	Acetyl-ornithine	0.996	0.089
Biogenic Amine	Putrescine	0.147	0.005
Biogenic Amine	alpha-Aminoadipic acid	1.240	0.024
Biogenic Amine	Taurine	66.405	2.030
Biogenic Amine	trans-Hydroxyproline	7.582	0.356
Biogenic Amine	Creatinine	71.029	1.459

Biogenic Amine	Serotonin	0.950	0.053
Biogenic Amine	Asymmetric dimethylarginine	0.449	0.009
Biogenic Amine	Total dimethylarginine	1.272	0.023
Biogenic Amine	Kynurenine	2.448	0.069
Biogenic Amine	Spermidine	0.241	0.003
Biogenic Amine	Spermine	0.174	0.002
Biogenic Amine	Sarcosine	0.943	0.052
Fatty Acid	Butyric acid	0.926	0.035
Fatty Acid	Propionic acid	0.899	0.025
Fatty Acid	Isobutyric acid	1.146	0.035
Organic Acid	Lactic acid	1,530.651	48.528
Organic Acid	beta-Hydroxybutyric acid	93.374	10.195
Organic Acid	alpha-Ketoglutaric acid	8.322	0.183
Organic Acid	Citric acid	107.836	3.257
Organic Acid	HPHPA	0.144	0.018
Organic Acid	p-Hydroxyhippuric acid	0.067	0.006
Organic Acid	Succinic acid	1.944	0.071
Organic Acid	Fumaric acid	1.189	0.043
Organic Acid	Pyruvic acid	42.049	2.075
Organic Acid	Hippuric acid	6.603	0.530
Organic Acid	Methylmalonic acid	0.164	0.012
Organic Acid	Homovanillic acid	0.047	0.002
Organic Acid	Indole acetic acid	1.934	0.132
Organic Acid	Uric acid	283.217	7.316
Other	Creatine	24.068	1.242
Other	Betaine	35.324	0.980
Other	Choline	9.446	0.214
Other	Trimethylamine N-oxide	6.822	1.133
Other	Methylhistidine	11.152	0.856
Other	Glucose	4,780.222	73.357
Glycerophospholipid	LYSOC14:0	4.156	0.118
Glycerophospholipid	LYSOC16:1	1.729	0.055

Glycerophospholipid	LYSOC16:0	51.435	1.264
Glycerophospholipid	LYSOC17:0	1.002	0.033
Glycerophospholipid	LYSOC18:2	19.399	0.811
Glycerophospholipid	LYSOC18:1	13.533	0.430
Glycerophospholipid	LYSOC18:0	14.742	0.429
Glycerophospholipid	LYSOC20:4	3.269	0.125
Glycerophospholipid	LYSOC20:3	0.869	0.045
Glycerophospholipid	LYSOC24:0	0.086	0.002
Glycerophospholipid	LYSOC26:1	0.077	0.002
Glycerophospholipid	LYSOC26:0	0.492	0.015
Glycerophospholipid	LYSOC28:1	0.304	0.011
Glycerophospholipid	LYSOC28:0	0.532	0.016
Glycerophospholipid	14:1SMOH	6.887	0.194
Glycerophospholipid	16:1SM	16.340	0.397
Glycerophospholipid	16:0SM	110.123	2.365
Glycerophospholipid	16:1SMOH	4.169	0.110
Glycerophospholipid	18:1SM	10.988	0.299
Glycerophospholipid	PC32:2AA	6.192	0.178
Glycerophospholipid	18:0SM	25.214	0.602
Glycerophospholipid	20:2SM	0.661	0.020
Glycerophospholipid	PC36:0AE	1.446	0.036
Glycerophospholipid	PC36:6AA	1.013	0.042
Glycerophospholipid	PC36:0AA	6.973	0.165
Glycerophospholipid	22:2SMOH	14.306	0.362
Glycerophospholipid	22:1SMOH	16.090	0.361
Glycerophospholipid	PC38:6AA	74.907	2.832
Glycerophospholipid	PC38:0AA	3.242	0.111
Glycerophospholipid	PC40:6AE	4.311	0.152
Glycerophospholipid	24:1SMOH	2.775	0.064
Glycerophospholipid	PC40:6AA	19.714	0.749
Glycerophospholipid	PC40:2AA	0.433	0.014
Glycerophospholipid	PC40:1AA	0.316	0.011

Acylcarnitine	C0 (Carnitine)	35.751	0.899
Acylcarnitine	C2 (Acetylcarnitine)	7.834	0.267
Acylcarnitine	C3:1 (Propenoylcarnitine)	0.051	0.002
Acylcarnitine	C3 (Propionylcarnitine)	0.272	0.009
Acylcarnitine	C4:1 (Butenylcarnitine)	0.018	0.000
Acylcarnitine	C4 (butyrylcarnitine)	0.193	0.008
Acylcarnitine	C3-OH (hydroxyPropionylcarnitine)	0.028	0.001
Acylcarnitine	C5:1 (Tiglylcarnitine)	0.050	0.001
Acylcarnitine	C5 (Valerylcarnitine)	0.094	0.003
Acylcarnitine	C4-OH (C3-DC) (Hydroxybutyrylcarnitine)	0.045	0.002
Acylcarnitine	C6:1 (Hexenoylcarnitine)	0.026	0.001
Acylcarnitine	C6 (C4:1-DC) (Hexanoylcarnitine)	0.084	0.003
Acylcarnitine	C5-OH (C3-DC-M) (hydroxyvalerylcarnitine)	0.035	0.001
Acylcarnitine	C5:1-DC (Glutaconylcarnitine)	0.015	0.000
Acylcarnitine	C5-DC (C6-OH)(Glutarylcarnitine)	0.015	0.000
Acylcarnitine	C8 (Octanoylcarnitine)	0.177	0.011
Acylcarnitine	C5-M-DC (methylglutarylcarnitine)	0.025	0.001
Acylcarnitine	C9 (Nonaylcarnitine)	0.047	0.003
Acylcarnitine	C7-DC (pimelylcarnitine)	0.077	0.007
Acylcarnitine	C10:2 (decadienylcarnitine)	0.059	0.002
Acylcarnitine	C10:1 (Decenoylcarnitine)	0.269	0.009
Acylcarnitine	C10 (Decanoylcarnitine)	0.469	0.029
Acylcarnitine	C12:1 (Dodecenoylcarnitine)	0.007	0.000
Acylcarnitine	C12 (dodecanoylcarnitine)	0.110	0.006
Acylcarnitine	C14:2 (Tetradecadienylcarnitine)	0.050	0.003
Acylcarnitine	C14:1 (tetradecenoyl carnitine)	0.161	0.008
Acylcarnitine	C14 (tetradecanoylcarnitine)	0.044	0.002
Acylcarnitine	C12-DC (dodecanedioylcarnitine)	0.127	0.005
Acylcarnitine	C14:2-OH (hydroxytetradecadienylcarnitine)	0.013	0.000
Acylcarnitine	C14:1-OH (Hydroxytetradecenoyl carnitine)	0.021	0.001
Acylcarnitine	C16:2 (Hexadecadienylcarnitine)	0.013	0.001
Acylcarnitine	C16:1 (Hexadecenoylcarnitine)	0.050	0.002

Acylcarnitine	C16 (Hexadecanoylcarnitine)	0.117	0.004
Acylcarnitine	C16:2-OH (hydroxyhexadecadienylcarnitine)	0.010	0.000
Acylcarnitine	C16:1-OH (Hydroxyhexadecenoylcarnitine)	0.015	0.000
Acylcarnitine	C16-OH (hydroxyhexadecanoylcarnitine)	0.009	0.000
Acylcarnitine	C18:2 (Octadecadienylcarnitine)	0.056	0.002
Acylcarnitine	C18:1 (Octadecenoylcarnitine)	0.159	0.005
Acylcarnitine	C18 (Octadecanoylcarnitine)	0.048	0.002

Data presented as mean ± standard error.
